# Donor hearts in the Sydney Heart Bank: reliable control but is it ‘normal’ heart?

**DOI:** 10.1007/s12551-020-00740-2

**Published:** 2020-07-20

**Authors:** Steven Marston, Adam Jacques, Christopher Bayliss, Emma Dyer, Massimiliano Memo, Maria Papadaki, Andrew Messer

**Affiliations:** 1 NHLI, Imperial College London, London, W12 0NN UK; 2 Cardiovascular Division, Imperial Centre for Translational and Experimental Medicine, Hammersmith Campus Du Cane Road, London, W12 0NN UK; 3 Cardiology Department, St Peters Hospital, Chertsey, KT16 0PZ UK; 4 ClinicalTrials, Guys and St Thomas Hospital Trust, London, SE1 9RT UK; 5 Intensive Care Unit, Royal Surrey County Hospital, Guildford, GU2 7XX UK; 6 Omega Biotek, E3 2UL, London, UK; 7 Department of Cell and Molecular Physiology, Loyola University Chicago, Chicago, IL USA

**Keywords:** Normal heart, Human myocardium, Donor heart, Contractility, Sydney Heart Bank, In vitro motility, Troponin I phosphorylation

## Abstract

Human heart samples from the Sydney Heart Bank have become a de facto standard against which others can be measured. Crucially, the heart bank contains a lot of donor heart material: for most researchers this is the hardest to obtain and yet is necessary since we can only study the pathological human heart in comparison with a control, preferably a normal heart sample. It is not generally realised how important the control is for human heart studies. We review our studies on donor heart samples. We report the results obtained with 17 different donor samples collected from 1994 to 2011 and measured from 2005 to 2015 by our standard methodology for in vitro motility and troponin I phosphorylation measurements. The donor heart sample parameters are consistent between the hearts, over time and with different operators indicating that Sydney Heart Bank donor hearts are a valid baseline control for comparison with pathological heart samples. We also discuss to what extent donor heart samples are representative of the normal heart.

**Electronic supplementary material:**

The online version of this article (10.1007/s12551-020-00740-2) contains supplementary material, which is available to authorized users.

## Introduction

It is a great pleasure to contribute to this special issue celebrating the achievements of Cris Dos Remedios and to celebrate his unique creation— the Sydney Heart Bank.

This story starts in Berlin in September 2000. I had known Cris as a biophysicist for as long as I can remember. The European Muscle Congress was held in Berlin for the first time in 2000—an age when PowerPoint was a new and risky mode of presentation. After the meeting finished, we found ourselves at a loose end waiting for evening flights and trains and went for a late lunch at the Potsdamer Platz. During our conversation, Cris revealed that he had been collecting human hearts from transplant operations in Sydney.

This was a great surprise to me and a pleasant one as well. A couple of years prior, I had started a project to apply our in vitro motility assay to the contractile proteins from human hearts. Ian Purcell, then an MD student, had been able to obtain a small amount of fresh heart tissue from transplant operations at the Freeman Hospital in Newcastle. We had a few end-stage failing samples and one donor sample. Cris said that he had dozens of samples, including donor hearts already and was continuously adding to the collection; moreover, he was willing to supply such samples to anyone who could make good use of them.

We immediately came to an agreement to work on these heart samples with our in vitro assays, and a month later, Cris sent us 5 1-g vials each of 5 donor hearts and 5 end-stage failing hearts. These were the basis of Andrew Messer’s thesis and many publications since.

The samples that Cris provided then and later were essential for the work we did for the next 15 years. The key merits of the Sydney Heart Bank are that the material is abundant, both in quantity per heart and number of hearts, that the collection regime is of the highest quality with very prompt freezing and storing in liquid nitrogen and that it is shared with the scientific community worldwide. This last point is crucial: there are a significant number of reports on human heart tissue, but they mainly use ‘private’ tissue collections; consequently, there is no way of independently checking whether such results are valid—a fundamental requirement in any scientific investigation.

Cris’ generosity in sharing his samples is outstanding, such that his samples have become a sort of de facto standard against which others can be measured. This has been well documented in recent publications (dos Remedios et al. 2017; Lal et al. 2015; Li et al. 2019) Crucially, his heart bank contains a lot of donor heart material: for most researchers, this is the hardest to obtain and yet is necessary since we can only study the pathological human heart in comparison with a control, preferably a normal heart sample. Cris’ donor hearts are such a control, and this review will be devoted to the studies on donor heart samples since it is not generally realised how important the control is for human heart studies.

We report the results obtained with 17 different donor samples collected from 1994 to 2011 and measured from 2005 to 2015 by our standard methodology for in vitro motility and troponin I phosphorylation measurements. The donor heart sample parameters are consistent between the hearts, over time and with different operators indicating that Sydney Heart Bank donor hearts are a valid baseline control for comparison with pathological heart samples.

## Materials and methods

Tissue samples were supplied by Prof. C Dos Remedios, University of Sydney, Australia. The protocols for tissue collection are well established (Lal et al. 2015), and the methods introduced at the Sydney Heart Bank—rapid freezing and storage in liquid nitrogen—have been followed by most other laboratories. Samples from the Sydney Heart Bank were shipped in liquid nitrogen and stored in liquid nitrogen for up to 11 years before use. Ethical approval was obtained from The Royal Brompton and Harefield NHS Trust, London, and St Vincent’s Hospital, Sydney. The investigation conformed with the principles outlined in the Declaration of Helsinki.

Donor myocardium was obtained from patients with brain stem death from a variety of causes who were assessed as potential heart donors, but their organs were subsequently rejected on technical grounds or because no suitable transplant recipient was found. The donors had no history of cardiac disease, a normal cardiac examination, a normal ECG and normal cardiac function on transthoracic echocardiography performed within 24 h of heart explantation. Myocardial samples were snap frozen in liquid nitrogen and stored for later analysis.

Proteins extracted from human heart donor samples were investigated in two assays. Troponin was isolated from 100 mg of human heart tissue by crushing the frozen tissue in a liquid nitrogen-cooled percussion mortar and rapidly extracting washed myofibrils. Myofibrils were dissolved in sample buffer, and the phosphospecies of troponin I were separated by phosphate affinity SDS-PAGE and identified by western blotting with an anti-cTnI monoclonal antibody as describe by Messer et al. (2009). Pure troponin was extracted from the myofibrils using an anti-cTnI antibody affinity column as described by Messer et al. (2007). Thin filaments were reconstituted with the human troponin, recombinant alpha-tropomyosin and skeletal muscle actin. The movement of thin filaments over a bed of immobilised skeletal muscle HMM and its regulation by Ca^2+^was visualised by the in vitro motility assay (Fraser and Marston 1995; Marston 2003; Marston et al. 1996). Ca^2+^-control of fraction motile and sliding speed was measured. Ca^2+^-sensitivity was determined by a curve fit of the data to the Hill equation. These methods were standard throughout the 12 years of this investigation.

Historic values from these assays were obtained from the PhD theses of Adam Jacques, Andrew Messer, Emma Dyer, Christopher Bayliss, Massimiliano Memo and Mary Papadaki, since these contained full details not present in their published papers, plus recent papers by Messer et al. (Bayliss 2011; Dyer 2008; Jacques 2012; Memo 2012; Messer 2007; Papadaki 2015).

## Results

I found 88 measurements of Ca^2+^-sensitivity from 17 donor samples with accompanying measurements of troponin I phosphorylation for each sample, measured over a period of 12 years. The results are shown in Supplementary Table 1. Many samples were measured multiple times (e.g. 2.149, 7 values; 4.104, 8 values; 5.089, 9 values; 5.090, 12 values; 5.126, 8 values; 6.008, 10 values; 7.08, 8 values).

Although the cause of brain death in these patients varied, time on life support varied from 20 to 135 h and times in cardioplegia also varied, non-failing donor heart muscle samples showed very similar motility parameters and phosphorylation levels.

**Fig. 1:**
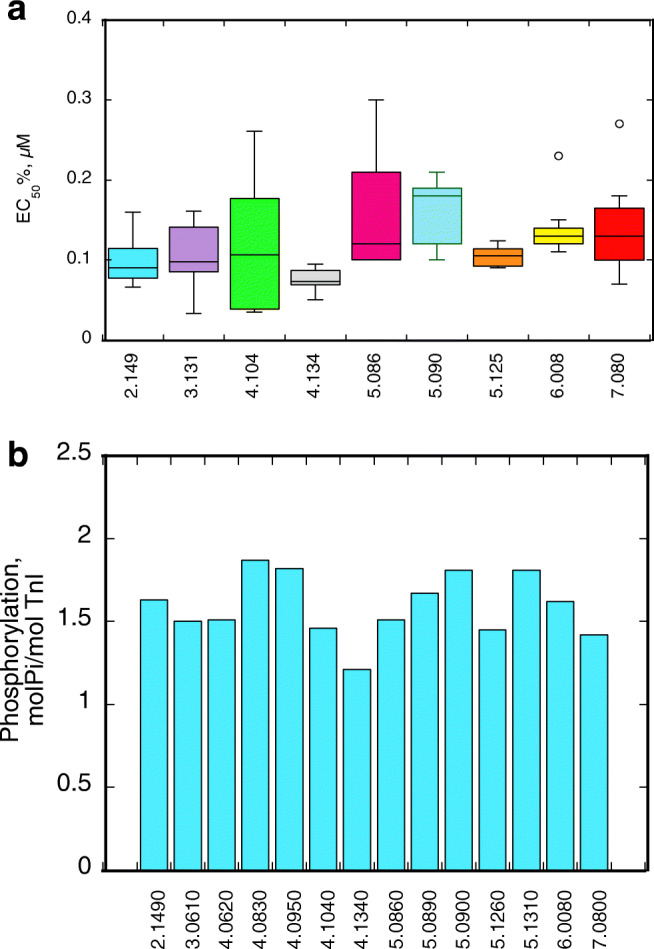
**a** Box and whisker plot of EC_50_ for Ca^2+^-activation of fraction of filaments motile measured in different donor samples by in vitro motility assay. Only the samples with 5 or more replicates are shown here (see Supplementary Table 1). The mean of all values is 0.137 μM ± .007 (SEM), 82 points. **b** Level of troponin I phosphorylation measured in 14 donor samples by phosphate affinity SDS-PAGE. The mean of these values is 1.59 molPi/molTnI ± 0.051 (SEM)

The variabilities between reported values do not correlate with the sample nor does it correlate with the operator. A statistical analysis of Messer’s data (Messer et al. 2007) shows that there was as much variability between 5 separate troponin preparations made from one muscle sample as between troponin made from six different muscle samples. This applies to both Ca^2+^-sensitivity of fraction motile and the level of TnI phosphorylation (although the latter had a smaller range of variation) as illustrated in Fig. 1. We also note that most of these samples have been used as control by other research groups measuring different properties of heart muscle as listed in Supplementary Table 1.

At the bottom of the table, we show results obtained with donor samples from other sources that were also rapidly frozen in liquid nitrogen: Ian Purcell’s sample from 1999 (Knott et al. 2002; Purcell et al. 1999) and recent samples collected by Ken Campbell, Kentucky University (Messer et al. 2016; Vikhorev et al. 2017). It is clear that these samples are similar to the Sydney Heart Bank samples when assayed in the same way.

## Discussion

Donor samples have been used as controls for studies on human heart for many years on the assumption that they are uniform without objective evidence that this was so. Operationally, all researchers have found that pathological material, be it failing heart, or familial heart diseases (hypertrophic cardiomyopathy, dilated cardiomyopathy) with known mutations, have clear and reproducible differences from donor heart, implicitly indicating that the donor heart is an acceptable control.

Our extensive measurements of myofilament Ca^2+-^sensitivity using the same techniques with many samples over many years clearly demonstrates that donor samples from the Sydney Heart Bank do indeed have uniform properties. Therefore, it is valid to use these samples as controls for comparative experiments (Messer et al. 2007), and it is also worth noting that other laboratories have collected donor heart tissue following the protocols established by the Sydney Heart Bank and come up with similar results.

Of course, it is entirely possible that when other properties of the samples are measured, variable results might be obtained from different sample donor samples. However, most studies have used several donor samples, and no such variability has been reported. Supplementary Table 1 lists other papers that use the samples we studied, and dos Remedios et al. list many more (dos Remedios et al. 2017). There are a few objective tests of whether preservation is adequate. RNA and protein degradation is an obvious way to detect a ‘bad’ sample. A particularly sensitive test is the level of protein phosphorylation, since, counter-intuitively, if tissue is not frozen soon enough or thaws out later, the level of TnI phosphorylation increases (Cai et al. 2018).

Despite the uniformity of donor samples, the question of whether such samples are normal remains to be addressed. The definition of a normal heart is itself difficult since the heart is such a dynamic organ. The resting heart (50–70 bpm) is the nearest to a standard state as could be defined. Applying such a criterion to the Sydney Heart Bank samples would suggest they are unlikely to be normal. The cause of brain death in these patients varied, time on life support varied from 20 to 135 h and times in cardioplegia also varied (Smith 2004). The argument could be made that all these samples are abnormal and that the uniformity of properties represents a common abnormality. The subject was much debated a few years ago, but not resolved, and it seems the two sides in the argument have agreed to differ (Jweied et al. 2007; Marston and deTombe 2008): I have not found any further studies that address this question since the work of Jweid in 2007.

It is well known that subarachnoid haemorrhage, stroke and trauma all cause markedly elevated catecholamine levels, and this may be increased again at the time of organ harvest (Fitzgerald et al. 1995); therefore, our samples may have abnormally high PKA phosphorylation levels. Protocols for organ harvesting are not available for Sydney Heart Bank samples and may have varied over the years anyway. Explanted hearts are usually maintained with inotropes which would tend towards a high level of phosphorylation of TnI and MyBP-C.

The ‘normality’ of Sydney Heart Bank samples is essentially unknowable. Nevertheless, several experiments indicate that abnormal phosphorylation levels may not be an issue for Sydney Heart Bank samples. Notably, the levels of troponin I and troponin T phosphorylation we found in mouse and guinea pig heart that had been rapidly frozen immediately after schedule 1 euthanasia without stimulation are the same as in donor heart muscle samples. In all three cases, only about 60% of the PKA sites on troponin I are phosphorylated which means that adrenergic activation is not maximal. In the mice, isoprenaline was shown to initiate an inotropic response with further phosphorylation (Pi et al. 2003; Pi et al. 2002), and further PKA phosphorylation decreased Ca^2+^-sensitivity in both human donor samples and mouse (Hamdani et al. 2009; van der Velden et al. 2003.; Wolff et al. 1996). Thus, on the basis of these rather limited criteria, Sydney Heart Bank samples may be representative of the normal heart muscle at rest.

## Electronic supplementary material

ESM 1 (DOCX 28 kb).
