# Supplementary material for: Donor hearts in the Sydney Heart Bank: reliable control but is it ‘normal’ heart?
Source: Biophys Rev. 2020 Jul 20;12(4):799–803. doi: 10.1007/s12551-020-00740-2 (PMC7429572; doi:10.1007/s12551-020-00740-2)
Supplement: Supplementary file 1 — (DOCX 28 kb). [file 12551_2020_740_MOESM1_ESM.docx]

**SUPPLEMENT**

Steven Marston, Adam Jacques, Christopher Bayliss, Emma Dyer, Massimiliano Memo, Maria Papadaki and Andrew Messer

Donor hearts in the Sydney Heart Bank: reliable control but is it ‘normal ‘ heart ?

**SUPPLEMENTARY TABLE 1**

Values for EC_50_ for Ca^2+^ regulation of fraction filaments motile and sliding speed and maximum sliding speed determined by the *in vitro* motility assay plus measurement of phosphorylation level using phosphate affinity SDS-PAGE for donor heart samples from the Sydney heart bank. Origin of data is indicated in the first column, Sample ID, sex, age and cause of death is indicated, together with date of collection and date of measurement. Samples were collected over a 20 year period and were measured 1-9 years after collection.

SAH sub-arachnoid haemorrhage

ICH intracranial haemorrhage

HBI hypoxic brain injury

MCA middle cerebral artery

TABLE 1

| source | Sydney Heart Bank ID | M/F | age | Cause of death | Date collected | Date measured | EC50 v, µM | EC50 %, µM | Sliding Velocity at pCa 5.5, µm/s | Phosphorylation of TnI , MolP/molTnI | Papers that use this sample |
| --- | --- | --- | --- | --- | --- | --- | --- | --- | --- | --- | --- |
|  |  |  |  |  |  |  |  |  |  |  |  |
| Jacques thesis | 2.149 | M | 44 | SAH | 1997 | 2006 | .16 | .08 | 4.47 | 1.63 | 1, 2, **12** |
| Jacques thesis | 2.149 | M | 44 | SAH | 1997 | 2006 | .12 | .09 | 4.14 |  |  |
| Jacques thesis | 2.149 | M | 44 | SAH | 1997 | 2006 |  | .16 | 3.86 |  |  |
| Messer Thesis | 2.149 | M | 44 | SAH | 1997 | 2005 | .344 | .075 | 4.34 |  |  |
| Messer Thesis | 2.149 | M | 44 | SAH | 1997 | 2005 | .112 | .066 | 4.97 |  |  |
| Messer Thesis | 2.149 | M | 44 | SAH | 1997 | 2005 | .217 | .134 | 3.98 |  |  |
| Messer Thesis | 2.149 | M | 44 | SAH | 1997 | 2005 | .112 | .095 | 4.49 |  |  |
| Jacques thesis | 3.061 | F | 34 | cerebral tumor | 2000 | 2006 | .12 | .07 | 3.39 | 1.50 | 10, **12, 16** |
| Messer Thesis | 3.061 | F | 34 | cerebral tumor | 2000 | 2005 | .337 | .128 | 4.24 |  |  |
| Messer Thesis | 3.064 | M | 54 | Trauma | 2000 | 2005 | .152 | .060 | 3.68 |  | 1, 3, 10, **12** |
| Jacques thesis | 3.073 | F | 41 | SAH | 2000 | 2006 | .19 | .05 | 3.94 | 1.54 | 1, 2, **12** |
| Messer Thesis | 3.073 | F | 41 | SAH | 2000 | 2005 | .195 | .080 | 3.19 |  |  |
| Messer Thesis | 3.131 | M | 51 | stroke | 2002 | 2005 | .351 | .085 | 3.29 |  | 1, 4, **12, 13, 16** |
| Messer Thesis | 3.131 | M | 51 | stroke | 2002 | 2005 | .321 | .161 | 4.63 |  |  |
| Messer Thesis | 3.131 | M | 51 | stroke | 2002 | 2005 | .129 | .098 | 4.016 |  |  |
| Messer Thesis | 3.131 | M | 51 | stroke | 2002 | 2005 | .428 | .141 | 4.06 |  |  |
| Messer Thesis | 3.131 | M | 51 | stroke | 2002 | 2005 | .169 | .033 | 3.97 |  |  |
| Dyer Thesis | 4.062 | M | 55 | gradeV SAH | 2004 | 2009 | .066 | .201 | 2.8 | 1.51 | 3, **15, 18** |
| Memo Thesis | 4.062 | M | 55 | SAH | 2004 | 2012 |  |  |  |  |  |
| Memo Thesis | 4.083 | M | 53 | Aneurism | 2004 | 2012 |  | .19 | 2.89 | 1.87 | **18** |
| Memo Thesis | 4.083 | M | 53 | Aneurism | 2004 | 2012 |  | .20 | 2.73 |  |  |
| Memo Thesis | 4.083 | M | 53 | Aneurism | 2004 | 2012 |  | .14 | 2.84 |  |  |
| Dyer Thesis | 4.095 | F | 48 | gradeV SAH | 2004 | 2009 | .025 | .044 | 3.2 | 1.82 | 1, **15** |
| Memo Thesis | 4.095 | F | 48 | SAH | 2004 | 2012 |  |  |  |  |  |
| Dyer Thesis | 4.104 | F | 59 |  | 2005 | 2009 | .264 | .177 | 2.7 | 1.46 | 8, 10, **15** |
| Dyer Thesis | 4.104 | F | 59 |  | 2005 | 2009 |  | .261 | 3.6 |  |  |
| Dyer Thesis | 4.104 | F | 59 |  | 2005 | 2009 |  | .039 | 2.8 |  |  |
| Dyer Thesis | 4.104 | F | 59 |  | 2005 | 2009 |  | .143 | 2.6 |  |  |
| Dyer Thesis | 4.104 | F | 59 |  | 2005 | 2009 |  | .035 | 2.8 |  |  |
| Dyer Thesis | 4.104 | F | 59 |  | 2005 | 2009 |  | .070 | 2.7 |  |  |
| Jacques thesis | 4.134 | F | 45 | ICH | 2005 | 2006 | .19 | .05 | 3.64 | 1.21 | **16** |
| Messer Thesis | 4.134 | F | 45 | ICH | 2005 | 2005 | .295 | .095 | 3.50 |  |  |
| Messer Thesis | 4.134 | F | 45 | ICH | 2005 | 2005 | .239 | .073 | 5.29 |  |  |
| Messer Thesis | 4.134 | F | 45 | ICH | 2005 | 2005 | .101 | .087 | 3.80 |  |  |
| Messer Thesis | 4.134 | F | 45 | ICH | 2005 | 2005 | .151 | .069 | 4.34 |  |  |
| Bayliss Thesis | 5.054 | M | 27 | SAH | 2006 | 2011 |  |  |  | 1.92 | 8, **12** |
| Bayliss Thesis | 5.086 | M | 29 | HBI | 2007 | 2011 |  | .33 | 2.14 | 1.51 | 6, 7, 8, **12** |
| Bayliss Thesis | 5.086 | M | 29 | HBI | 2007 | 2011 |  | .21 | 1.99 |  |  |
| Bayliss Thesis | 5.086 | M | 29 | HBI | 2007 | 2011 |  | .30 | 2.38 |  |  |
| Bayliss Thesis | 5.086 | M | 29 | HBI | 2007 | 2011 |  | .10 | 1.6 |  |  |
| Memo Thesis | 5.086 | M | 29 | HBI | 2007 | 2012 |  | 0.12 | 3.41 |  |  |
| Memo Thesis | 5.086 | M | 29 | HBI | 2007 | 2012 |  | 0.10 | 3.02 |  |  |
| Bayliss Thesis | 5.089 | F | 48 | SAH | 2007 | 2011 |  |  |  | 1.67 | 8, **12, 18** |
| Memo Thesis | 5.089 | F | 48 | SAH | 2007 | 2012 | .09 | .13 | 2.35 |  |  |
| Memo Thesis | 5.089 | F | 48 | SAH | 2007 | 2012 | .06 | .16 | 2.52 |  |  |
| Bayliss Thesis | 5.090 | F | 42 | SAH | 2008 | 2011 |  | 0.1 | 2.25 | 1.81 | 11, 20, **12** |
| Bayliss Thesis | 5.090 | F | 42 | SAH | 2008 | 2011 |  | 0.19 | 2.77 |  |  |
| Bayliss Thesis | 5.090 | F | 42 | SAH | 2008 | 2011 |  | 0.18 | 2.28 |  |  |
| Bayliss Thesis | 5.090 | F | 42 | SAH | 2008 | 2011 |  | 0.21 | 2.68 |  |  |
| Bayliss Thesis | 5.090 | F | 42 | SAH | 2008 | 2011 |  | 0.1 | 2.24 |  |  |
| Bayliss Thesis | 5.090 | F | 42 | SAH | 2008 | 2011 |  | .36 | 3.3 |  |  |
| Bayliss Thesis | 5.090 | F | 42 | SAH | 2008 | 2011 |  | .18 | 3.06 |  |  |
| Bayliss Thesis | 5.090 | F | 42 | SAH | 2008 | 2011 |  | .21 | 3.3 |  |  |
| Memo Thesis | 5.090 | F | 42 | SAH | 2008 | 2012 |  | .13 | 3.2 |  |  |
| Memo Thesis | 5.090 | F | 42 | SAH | 2008 | 2012 |  | .11 | 2.51 |  |  |
| Memo Thesis | 5.090 | F | 42 | SAH | 2008 | 2012 |  | .18 | 2.55 |  |  |
| Memo Thesis | 5.090 | F | 42 | SAH | 2008 | 2012 |  | .19 | 2.87 |  |  |
| Messer (17) | 5.126 | F | 55 | SAH | 2009 | 2014 |  | 0.09 |  | 1.45 | 11,20, **12, 17** |
| Messer (17) | 5.126 | F | 55 | SAH | 2009 | 2014 |  | .113 |  |  |  |
| Messer (17) | 5.126 | F | 55 | SAH | 2009 | 2014 |  | .115 |  |  |  |
| Messer (17) | 5.126 | F | 55 | SAH | 2009 | 2014 |  | .090 |  |  |  |
| Messer (17) | 5.126 | F | 55 | SAH | 2009 | 2014 |  | .094 |  |  |  |
| Messer (17) | 5.126 | F | 55 | SAH | 2009 | 2014 |  | .100 |  |  |  |
| Messer (17) | 5.126 | F | 55 | SAH | 2009 | 2014 |  | .111 |  |  |  |
| Messer (17) | 5.126 | F | 55 | SAH | 2009 | 2014 |  | .124 |  |  |  |
| Bayliss Thesis | 5.131 | F | 42 | Aneurism | 2009 | 2011 | .10 | .08 |  | 1.81 | 20, **12** |
| Bayliss Thesis | 5.131 | F | 42 | Aneurism | 2009 | 2011 | .14 | .12 |  |  |  |
| Bayliss Thesis | 5.131 | F | 42 | Aneurism | 2009 | 2011 | .25 | .16 |  |  |  |
| Bayliss Thesis | 5.131 | F | 42 | Aneurism | 2009 | 2011 | .14 | ..12 |  |  |  |
| Bayliss Thesis | 5.131 | F | 42 | Aneurism | 2009 | 2011 | .15 | .10 |  |  |  |
| Papadaki Thesis | 6.008 | M | 40 | MCA infarct | 2009 | 2015 | .08 | .12 |  | 1.62 | 6, 9, 20, **12, 19** |
| Papadaki Thesis | 6.008 | M | 40 | MCA infarct | 2009 | 2015 | .09 | .15 |  |  |  |
| Papadaki Thesis | 6.008 | M | 40 | MCA infarct | 2009 | 2015 | .09 | .13 |  |  |  |
| Papadaki Thesis | 6.008 | M | 40 | MCA infarct | 2009 | 2015 | .29 | .23 |  |  |  |
| Papadaki Thesis | 6.008 | M | 40 | MCA infarct | 2009 | 2015 | .12 | .11 |  |  |  |
| Papadaki Thesis | 6.008 | M | 40 | MCA infarct | 2009 | 2015 | .18 | .14 |  |  |  |
| Papadaki Thesis | 6.008 | M | 40 | MCA infarct | 2009 | 2015 | .10 | .14 |  |  |  |
| Papadaki Thesis | 6.008 | M | 40 | MCA infarct | 2009 | 2015 | .14 | .13 |  |  |  |
| Papadaki Thesis | 6.008 | M | 40 | MCA infarct | 2009 | 2015 | .12 | .12 |  |  |  |
| Papadaki Thesis | 6.008 | M | 40 | MCA infarct | 2009 | 2015 | .11 | .11 |  |  |  |
| Papadaki Thesis | 7.080 | F | 55 | SAH | 2010 | 2015 | .14 | .18 |  | 1.42 | **19** |
| Papadaki Thesis | 7.080 | F | 55 | SAH | 2010 | 2015 | .12 | .15 |  |  |  |
| Mary Thesis | 7.080 | F | 55 | SAH | 2010 | 2015 | .13 | .27 |  |  |  |
| Papadaki Thesis | 7.080 | F | 55 | SAH | 2010 | 2015 | .11 | .08 |  |  |  |
| Papadaki Thesis | 7.080 | F | 55 | SAH | 2010 | 2015 | .11 | .13 |  |  |  |
| Papadaki Thesis | 7.080 | F | 55 | SAH | 2010 | 2015 | .07 | .07 |  |  |  |
| Papadaki Thesis | 7.080 | F | 55 | SAH | 2010 | 2015 | .11 | .12 |  |  |  |
|  |  |  |  |  |  |  |  |  |  |  |  |
| Jacques Thesis | Freeman Hospital, Newcastle | M | 51 | stroke | 1997 | 2001 |  | .12 |  |  | **12** |
| Kenneth Campbell | Univ Kentucky  24713 | F | 47 | Head trauma |  | 2017 |  | .067 |  |  | **14, 17** |
|  |  |  |  |  |  |  |  |  |  |  |  |
| Mean |  |  |  |  |  |  |  | **.131** | **3.27** | **1.59** |  |
| SEM |  |  |  |  |  |  |  | **.007** | **.114** | **.051** |  |
| n |  |  |  |  |  |  |  | **82** | **53** | **14** |  |

**REFERENCES BOLD** from this lab

1 (Kong et al. 2010)

2 (Zhang et al. 2012)

3 (Kagemoto et al. 2018)

4 (Kötter et al. 2013)

**5 (Hoskins et al. 2010)**

6 (Bollen et al. 2017)

7 (Mamidi et al. 2017)

8 (Mollova et al. 2013)

9 (McNamara et al. 2017)

**10 (Jacques et al. 2009)**

11 (Tucholski et al. 2020)

**12 (Bayliss et al. 2012)**

**13 (Knott et al. 2002)**

**14 (Vikhorev et al. 2017)**

**15 (Dyer et al. 2009)**

**16 (Messer et al. 2007)**

**17 (Messer et al. 2016)**

**18 (Memo et al. 2013)**

**19 (Papadaki et al. 2015)**

20… (Sanchez-Alonso et al. 2016)

**PhD Theses:**

Adam Jacques: (Jacques 2012)

Andrew Messer: (Messer 2007)

Emma Dyer: (Dyer 2008)

Massimiliano Memo: (Memo 2012)

Christopher Bayliss: (Bayliss 2011)

Maria Papadaki: (Papadaki 2015)

REFERENCES

Bayliss C (2011) DYSFUNCTION OF CONTRACTILE PROTIENS IN HYPERTROPHIC CARDIOMYOPATHY. Imperial College London

Bayliss CR et al. (2012) Myofibrillar Ca2+-Sensitivity Is Uncoupled From Troponin I Phosphorylation In Hypertrophic Obstructive Cardiomyopathy Due To Abnormal Troponin T. Cardiovasc Res 97:500-508 doi:10.1093/cvr/cvs322

Bollen IAE et al. (2017) Myofilament Remodeling and Function Is More Impaired in Peripartum Cardiomyopathy Compared with Dilated Cardiomyopathy and Ischemic Heart Disease The American journal of pathology 187:2645-2658 doi:10.1016/j.ajpath.2017.08.022

Dyer E (2008) A Functional Investigation of Mutations Associated with Hypertrophic and Dilated Cardiomyopathy Doctoral Thesis, Imperial College London

Dyer E et al. (2009) Functional Analysis of a Unique Troponin C Mutation, Gly159Asp that Causes Familial Dilated Cardiomyopathy, Studied in Explanted Heart Muscle Circ Heart Fail 2:456-464 doi:10.1161/CIRCHEARTFAILURE.108.818237

Hoskins AC et al. (2010) Normal passive viscoelasticity but abnormal myofibrillar force generation in human hypertrophic cardiomyopathy J Mol Cell Cardiol 49:737-745 doi:10.1016/j.yjmcc.2010.06.006

Jacques A (2012) Hypertrophic and Dilated Cardiomyopathies, the relationship of phenotype to genotype. Imperial College London

Jacques A, Hoskins A, Kentish J, Marston SB (2009) From genotype to phenotype: a longitudinal study of a patient with hypertrophic cardiomyopathy due to a mutation in the MYBPC3 gene J Musc Res Cell Motil 29:239-246 doi:10.1007/s10974-009-9174-0

Kagemoto T et al. (2018) Sarcomeric Auto-Oscillations in Single Myofibrils From the Heart of Patients With Dilated Cardiomyopathy Circ: Heart Fail 11:e004333 doi:doi:10.1161/CIRCHEARTFAILURE.117.004333

Knott A, Purcell IF, Marston S (2002) In vitro motility analysis of thin filaments from failing and non-failing human hearts induces slower filament sliding and higher Ca2+-sensitivity J Mol Cell Cardiol 34:469–482

Kong SW et al. (2010) Heart Failure&#x2013;Associated Changes in RNA Splicing of Sarcomere Genes Circ: Cardiovasc Genet 3:138-146 doi:doi:10.1161/CIRCGENETICS.109.904698

Kötter S et al. (2013) Differential changes in titin domain phosphorylation increase myofilament stiffness in failing human hearts Cardiovascular Research 99:648-656 doi:10.1093/cvr/cvt144

Mamidi R et al. (2017) Dose-Dependent Effects of the Myosin Activator Omecamtiv Mecarbil on Cross-Bridge Behavior and Force Generation in Failing Human Myocardium Circ: Heart fail 10:e004257 doi:10.1161/CIRCHEARTFAILURE.117.004257

McNamara JW et al. (2017) MYBPC3 mutations are associated with a reduced super-relaxed state in patients with hypertrophic cardiomyopathy PloS one 12:e0180064-0180022 doi:10.1371/journal.pone.0180064

Memo M (2012) Molecular Mechanisms of Myopathies. Imperial College London

Memo M et al. (2013) Mutations in thin Filament Proteins that Cause Familial Dilated Cardiomyopathy Uncouple Troponin I Phosphorylation from Changes in Myofibrillar Ca2+-Sensitivity Cardiovasc Res 99:65-73 doi:10.1093/cvr/cvt071

Messer A (2007) Structural and functional polymorphisms of troponin in failing heart. PhD Thesis London

Messer A et al. (2016) Mutations in troponin T associated with Hypertrophic Cardiomyopathy increase Ca2+-sensitivity and suppress the modulation of Ca2+-sensitivity by troponin I phosphorylation Arch Biochem Biophys 601:113-120

Messer AE, Jacques AM, Marston SB (2007) Troponin phosphorylation and regulatory function in human heart muscle: Dephosphorylation of Ser23/24 on troponin I could account for the contractile defect in end-stage heart failure J Mol Cell Cardiol 42:247-259 doi:doi:10.1016/j.yjmcc.2006.08.017

Mollova M et al. (2013) Cardiomyocyte proliferation contributes to heart growth in young humans Proc Natl Acad Sci U S A 110:1446-1451 doi:10.1073/pnas.1214608110

Papadaki M (2015) The importance of uncoupling of troponin I phosphorylation from Ca2+ sensitivity in the pathogenesis of cardiomyopathy. Imperial College London

Papadaki M, Vikhorev PG, Marston SB, Messer AE (2015) Uncoupling of myofilament Ca2+ sensitivity from troponin I phosphorylation by mutations can be reversed by epigallocatechin-3-gallate Cardiovasc Res 108:99-110 doi:10.1093/cvr/cvv181

Sanchez-Alonso JL et al. (2016) Microdomain-Specific Modulation of L-Type Calcium Channels Leads to Triggered Ventricular Arrhythmia in Heart Failure Circ Res 119:944-955 doi:10.1161/CIRCRESAHA.116.308698

Tucholski T et al. (2020) Converging sarcomeric proteoform phenotypes in Human hypertrophic cardiomyopathy revealed by top-down proteomics Proc Natl Acad Sci U S A submitted

Vikhorev PG et al. (2017) Abnormal contractility in human heart myofibrils from patients with dilated cardiomyopathy due to mutations in TTN and contractile protein genes Sci Rep 7:14829 doi:10.1038/s41598-017-13675-8

Zhang P, Kirk JA, Ji W, dos Remedios CG, Kass DA, Van Eyk JE, Murphy AM (2012) Multiple Reaction Monitoring to Identify Site-Specific Troponin I Phosphorylated Residues in the Failing Human Heart Circulation 126:1828-1837 doi:10.1161/CIRCULATIONAHA.112.096388
